# Ovariectomy-Induced Reductions in Endothelial SK3 Channel Activity and Endothelium-Dependent Vasorelaxation in Murine Mesenteric Arteries

**DOI:** 10.1371/journal.pone.0104686

**Published:** 2014-08-08

**Authors:** Fui C. Yap, Mark S. Taylor, Mike T. Lin

**Affiliations:** Department of Physiology, University of South Alabama, Mobile, Alabama, United States of America; Temple University School of Medicine, United States of America

## Abstract

Mesenteric artery endothelium expresses both small (SK3)- and intermediate (IK1)-conductance Ca^2+^-activated K^+^ (K_Ca_) channels whose activity modulates vascular tone via endothelium-dependent hyperpolarization (EDH). Two other major endothelium-dependent vasodilation pathways utilize nitric oxide (NO) and prostacyclin (PGI_2_). To examine how ovariectomy (ovx) affects the basal activity and acetylcholine (ACh)-induced activity of each of these three pathways to vasorelaxation, we used wire myograph and electrophysiological recordings. The results from functional studies using isolated murine mesenteric arteries show that ovx reduces ACh-induced endothelium-dependent vasodilation due to decreased EDH and NO contributions, although the contribution of PGI_2_ is upregulated. Both endothelial SK3 and IK1 channels are functionally coupled to TRPV4 (transient receptor potential, vanilloid type 4) channels: the activation of TRPV4 channels activates SK3 and IK1 channels, leading to EDH-mediated vascular relaxation. The decreased EDH-mediated vasorelaxation in ovx vessels is due to reduced SK3 channel contribution to the pathway. Further, whole-cell recordings using dispersed endothelial cells also show reduced SK3 current density in ovx endothelial cells. Consequently, activation of TRPV4 channels induces smaller changes in whole-cell current density. Thus, ovariectomy leads to a reduction in endothelial SK3 channel activity thereby reducing the SK3 contribution to EDH vasorelaxation.

## Introduction

Vascular endothelial cells provide important regulatory mechanisms to the modulation of vascular tone. These endothelial vasoactive factors include both vasoconstrictors and vasodilators, and the contribution of ECs to vascular tone is the net effect of these vasoactive factors. An imbalance of vasoactive factors that generally leads to an enhanced vasoconstriction is called endothelial dysfunction [1]. The three major endothelium-dependent vasodilation pathways best characterized are: nitric oxide (NO), prostacyclin (PGI_2_), and endothelium-dependent hyperpolarization (EDH), with EDH-induced vasorelaxation being the least understood. Each of their contribution to vascular tone may change with physiological conditions, such as menopause, diabetes, and aging [2]–[4].

Recently several studies have begun to elucidate the underlying vasodilatory mechanism of EDH. Results from our group and others have shown unequivocal importance of endothelial small (SK3)- and intermediate (IK1)-conductance Ca^2+^-activated K^+^ channels in EDH-mediated relaxation [5], [6]. SK3 channels are likely distributed throughout the plasma membrane and their localization and trafficking may be linked to caveolae [7], [8]. On the other hand, the expression of IK1 may be more concentrated on endothelial projections, which protrude through the internal elastic lamina and electrically couple to vascular myocytes via gap junctions [9]. Sonkusare *et al.* has recently demonstrated that activating TRPV4 (transient receptor potential, vanilloid type 4) channels induces vasodilation in mesenteric arteries due to Ca^2+^ influx via these channels that leads to Ca^2+^-activation of either SK3 or IK1 channels, and activation of the EDH pathway [10].

The consequences of losing circulating ovarian hormones have been intensively studied, although the significance of hormone replacement therapy remains controversial. Previous studies have demonstrated beneficial neurological and cardiovascular effects of circulating ovarian hormones–such as neuronal protection from ischemic stroke, coronary artery diseases, and high blood pressure [11]–[14]. Of the female hormones produced from ovaries, estrogen is the most extensively studied and has been shown to play important roles in both the maintenance of normal vascular function and the prevention of endothelial dysfunction. Depending upon the vascular beds and the size of vessels, the mechanisms underlying estrogen-dependent regulation of vascular tone–hence cardiovascular protection–are different. For example, surgical removal of ovaries, or ovariectomy (ovx), which causes a rapid reduction in the plasma level of estrogen, shifts endothelial-dependent vasorelaxation toward EDH-mediated mechanisms in middle cerebral arteries [15]. In contrast, ovx reduces EDH contribution to vasorelaxation in mesenteric arteries [16].

In animal models, a preponderance of evidence has shown that estrogen, which induces vasodilation through direct modulation on both vascular smooth muscle cells and endothelial cells (ECs), may be the key to female cardioprotection [17]. However, despite findings in animal models showing estrogen’s cardiovascular protective effect, large-scale human studies have shown that the estrogen replacement therapy do not seem to warrant significant cardiovascular protection; the detrimental ramification of added estrogen, which may induce breast cancer growth, might in fact outweigh its beneficial effects [18], [19]. The disparity between animal and human studies are under vigorous re-evaluation; nevertheless, the mechanisms underlying the loss of cardiovascular protection following menopause remain elusive [10].

It has been shown in heterologous expression systems as well as in neurons that estrogen may either enhance or reduce SK3 channel expression levels [20], [21]. Because estrogen is only one of many hormones produced by the ovary, and losing the conglomerate of ovarian hormones may underlie the loss of cardiovascular protection, one of our main goals in the current study was to examine how ovx affects each of the three major endothelium-dependent vasorelaxation pathways. Utilizing a combination of functional and electrophysiological studies, we show that ovariectomy causes an increased PGI_2_ and decreased NO and EDH contribution to vasorelaxation in murine mesenteric arteries. Further examination of the endothelial SK3 and IK1 channels shows that although ECs from both animal groups express comparable IK1 channel current density, reduced SK3 channel current density leads to a reduction in EDH-mediated vasorelaxation in ovx mice.

## Methods

### Animals

All animal surgeries and experimental procedures were approved by the Institutional Animal Care and Use Committee of the University of South Alabama, and conducted according to the Guide to the Care and Use of Laboratory Animals of the National Institutes of Health. Two groups of adult C57BL/6J mice were used in the present study: un-operated control female mice and ovariectomized (ovx) mice. Ovariectomy was performed under general anesthesia using a mixture of ketamine and xylazine. Immediately post-operatively, animals were placed in a clean cage on top of a warming pad to recover and they were monitored until ambulatory. Mice received a subcutaneous injection of Buprenex as they were coming out of anesthesia and 1–2 days after surgery. Mice were checked daily after surgery and animals exhibiting complications or distress were euthanized.

Age-matched mice were weighed at 9–12 weeks of age, with half of them undergoing ovariectomy and the other half used as control. The weight of these mice prior to ovariectomy did not differ (P>0.05). These mice were weighed again 4–6 wks post surgery, right before they were sacrificed. On average, the weight of ovx mice was significantly heavier than that of control mice (ovx 33.5±1.9 g vs. control 23.4±1.0 g; n = 12; P<0.05). Moreover, a group of 6 sham-operated control mice, which were operated on but without the removal of ovaries, did not show a significant change in body weight from that of the control group (sham control 21.6±1.7 g; n = 6; P>0.05). These results are consistent with previous reports showing the loss of estrogen induces body weight gain [22].

Mice were euthanized with isoflurane overdose and tissues were collected and put on ice-cold buffer solution. First- and second-order mesenteric arteries were carefully dissected out free of surrounding tissues in ice-cold low Ca^2+^ HEPES solution containing (in mM): NaCl 134, KCl 6, MgCl_2_ 0.2, CaCl_2_ 0.1, glucose 10, and HEPES 10 (pH 7.4), and mounted in a wire myograph or digested enzymatically to obtain dispersed endothelial cells.

### Myography

Arteries mounted in wire myograph (Danish Myo Technology, DMT, Denmark), were bathed in 37°C bicarbonate-based physiological salt solution (PSS; in mM): NaCl 119, KCl 4.7, KH_2_PO_4_ 1.2, MgSO_4_ 1.2, CaCl_2_ 2, EDTA 0.026, glucose 10.5, and NaHCO_3_ 23, constantly bubbled with 95% O_2_ and 5% CO_2_. Vessels were equilibrated for 30 min and stretched to their optimal resting tension of ∼2 mN, as determined in previous study [5], followed by equilibration for another 10 min before the start of experiments. For cumulative concentration-response studies, arteries were bathed in different concentrations of phenylephrine (PE), followed by bath incubation in 60 mM KCl PSS containing (in mM): NaCl 59, KCl 64.7, KH_2_PO_4_ 1.2, MgSO_4_ 1.2, CaCl_2_ 2, EDTA 0.026, glucose 10.5, and NaHCO_3_ 23 at 37°C to obtain maximum tension. PE concentration-response curves were normalized to KCl-induced maximum force for each vessel. After bath washout for several times, vessels were pre-contracted with PE to ∼50% (EC_50_) of maximum tension and different cumulative concentrations of acetylcholine (ACh) were bath applied to determine its concentration responses.

For all other myography studies, following the same equilibration periods as described, arteries were precontracted with 3 µM PE (EC_80_), relaxed with subsequent addition of 1 µM ACh, followed by incubation in 60 mM KCl PSS. After several washes vessels were 50% pre-contracted with PE and selective blocker of endothelium-dependent relaxation pathways were used to study their preexisting vascular activity [23]. These blockers include: L-NAME (NG-nitro-L-arginine methyl ester, blocks nitric oxide production; 100 µM), indomethacin (blocks prostacyclin production; 10 µM), apamin (blocks SK channels; 300 nM) and tram34 (blocks IK1 channels; 1 µM). Vascular tension gradually increased in the presence of each of these blockers, and the time it took to reach a steady plateau was ∼15 and 10 min for L-NAME and indomethacin, respectively. Simultaneous blockade of SK and IK1 channels abolishes EDH-induced relaxation and in the presence of apamin and tram34, vascular tension increased to plateau in ∼8 min [24], [25]. TRPV4 channel modulators HC067047 (HC, blocks TRPV4 channels; 500 nM) and GSK1016790 (GSK, activates TRPV4 channels; 300 nM) were also used to study its contribution to vascular tone. 1 µM ACh was always added at the end of experiment and the ACh-induced vasorelaxation in the presence and absence of each inhibitor were compared. The preexisting activity of specific pathways to tone were determined from the relative contractile effects of inhibitors, expressed as percent increase in force relative to steady-state. The contributions of specific pathways to ACh-induced relaxation were determined by comparing ACh relaxations (change in force) before and after inhibitor treatments, and expressing the difference as percent of the control (before) response. [(ACh before inhibitor – after inhibitor)/before inhibitor)]×100%. Arteries that did not show ACh-induced endothelium-dependent vasorelaxation, hence indicating damage to the endothelium, were discarded. Myography data were both acquired and analyzed using LabChart 7 (DMT, Denmark).

### Cell Isolation

To obtain dispersed endothelial cells (ECs), cleaned mesenteric arteries were placed in 37°C HEPES solution containing (in mM): NaCl 55, Na-glutamate 80, KCl 5.9, MgCl_2_ 2, CaCl_2_ 0.1, glucose 10, and HEPES 10 (pH 7.3), with 0.5 mg/ml protease, 0.5 mg/ml elastase for 50 min, followed by additional 5 min in the same solution containing 0.5 mg/ml collagenase (modified from [10]). The tissue was then washed several times with ice-cold Ca^2+^ free HEPES solution and triturated with a fire-polished pasture pipette. Isolated ECs were kept in the ice-cold solution and recorded within 6 hours.

### Electrophysiology

Whole-cell voltage clamp recordings were performed on isolated ECs using an Axopatch 200B amplifier, Digidata 1322A, and data were acquired using PClamp 8 software (all from Molecular Devices, Sunnyvale, CA). Cells were clamped at their resting membrane potential and whole-cell currents were evoked every 30 s with a voltage protocol consisting of 3 segments: a 20 ms hyperpolarizing step for membrane capacitance measurement; a 200 ms voltage ramp from −80 to +60 mV; and a 100 ms step at +30 mV [7]. Currents were sampled at 2 kHz and filtered at 1 kHz, and normalized to membrane capacitance to obtain current densities.

Patch pipettes (3–5 MΩ) were filled with internal pipette solution contained (in mM): KCl 130, NaCl 10, EDTA 5, HEPES 10, MgCl_2_ 5.13, CaCl_2_ 1.01 (pH 7.2, 285 mOsm). The concentrations of free Mg^2+^ (1 mM) and Ca^2+^ (3 µM) were calculated using Patcher’s Power Tools (Department of Membrane Biophysics at MPI Biophysical Chemistry in Göttingen, Germany). For perforated patch recordings in current clamp mode, patch pipettes were back-filled with pipette solution containing amphotericin B (150 µg/ml), and access resistance was monitored with a hyperpolarizing pulse [7], [26]. External bath solution contained (in mM): NaCl 134, KCl 6, MgCl_2_ 1, CaCl_2_ 2, glucose 10, and HEPES 10 (pH 7.3, 280 mOsm). Osmolarity for all solutions was verified with Osmette III osmometer (Precision systems, Natick MA). Pharmacological agonists and antagonists of ion channels were perfused into the recording chamber via a gravity perfusion system. GSK (30 nM) and HC (500 nM) were used as TRPV4 channel agonist and antagonist, respectively. Tram34 (1 µM) and apamin (300 nM) were used to block IK1 and SK3 channels, respectively. Amphotericin B and GSK was obtained from Sigma-Aldrich (St. Louis, MO). HC was obtained from Tocris (Minneapolis, MN). Apamin was obtained from EMD Millipore (Philadelphia, PA). Tram34 was from Alomone Labs (Israel). All other chemicals were obtained from Fisher Scientific (Pittsburgh, PA).

### Data and analysis

Myography data were analyzed using LabChart (DMT, Denmark). IgorPro (WaveMetrics, Lake Oswego, OR) were used to analyze electrophysiological data and to prepare and plot all figures shown in this study. Averaged and normalized data are expressed as mean ± SEM. Paired two sample *t*-tests were used to determine significance of data from the same vessel or cell; ANOVA with Dunnett’s posthoc tests were used to determine significance among different groups of data. P<0.05 was considered significant.

## Results

### Phenylephrine and acetylcholine concentration-response curves with force myograph measurements using mesenteric arteries obtained from control and ovariectomized mice

**Figure 1:**
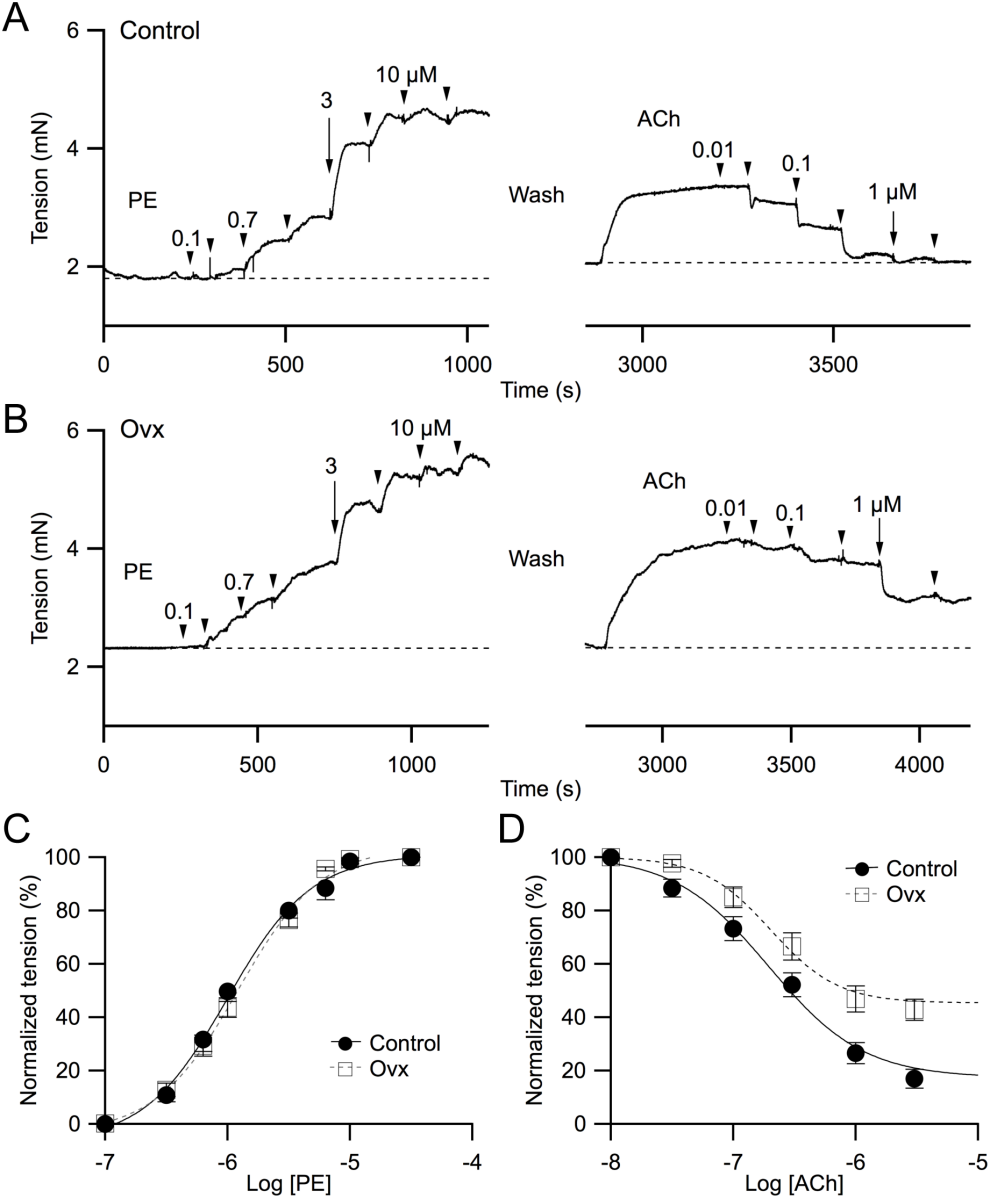
Ovariectomy reduces ACh-induced vasorelaxation. Representative force myograph traces showing isometric tension (mN) plotted against time (s) for both control (**A**) and ovariectomized (ovx; **B**) mesenteric vessels, and their concentration responses to both phenylephrine (PE; left panel) and acetylcholine (ACh; right panel). **A and B**: (left panel) Arrowheads and arrow markers show addition of different [PE] (from left to right: 0.1, 0.3, 0.7, 1, 3, 7, 10, and 30 µM); the arrow indicates the addition of 3 µM PE. (right panel) Following PE washout the vessels were ∼50% pre-contracted with PE and different [ACh] were added (markers from left to right: 0.01, 0.03, 0.1, 0.3, 1, 3 µM), with the arrow indicating 1 µM ACh. Normalized concentration-response of PE (**C**) and ACh (**D**) for both control (solid circles) and ovx (empty squares) vessels. Data were fitted with sigmoidal curves for both control (solid lines) and ovx (dotted lines) vessels. Both plots were normalized to the maximum PE-induced tension.

First- and second-order mesenteric arteries, obtained from age-matched female non-operated control and ovariectomized (ovx; 4–6 weeks post ovariectomy; see Methods for details) mice, were mounted in an isometric wire myograph system. Following equilibration for at least 30 min at 37°C vessels were stretched to their optimal isometric initial resting tension of ∼2 mN and a stable baseline was established for at least 10 min before experimentation. Bath applications of phenylephrine (PE) contracted both control and ovx mesenteric arteries in a concentration-dependent manner (Fig. 1 left). Normalized concentration-response curves (Figure 1C) showed no significant differences in the overall sensitivity to PE between control and ovx mice (EC_50_ values: control 1.0±0.1 µM vs. 1.2±0.1 µM; n = 10). Following bath washout, the same vessels were pre-contracted with PE to induce ∼50% increase in tension (EC_50_), followed by bath applications of different concentrations of acetylcholine (ACh), and the concentration-response curves of ACh-induced vasorelaxation for these vessels were also established (Fig. 1 right). ACh-induced vasorelaxation was normalized to PE-induced contraction and fitted with sigmoidal curves for both control and ovx groups. Despite having similar IC_50_ values (control: 0.21±0.01 µM vs. ovx: 0.20±0.02 µM; n = 10), maximal ACh-induced vasorelaxation was reduced in vessels isolated from ovx animals (tension at 1 µM: control: 26.5% vs. ovx 46.9%; Fig. 1D).

### ACh-induced vasorelaxation is diminished in mesenteric arteries obtained from ovx mice

**Figure 2:**
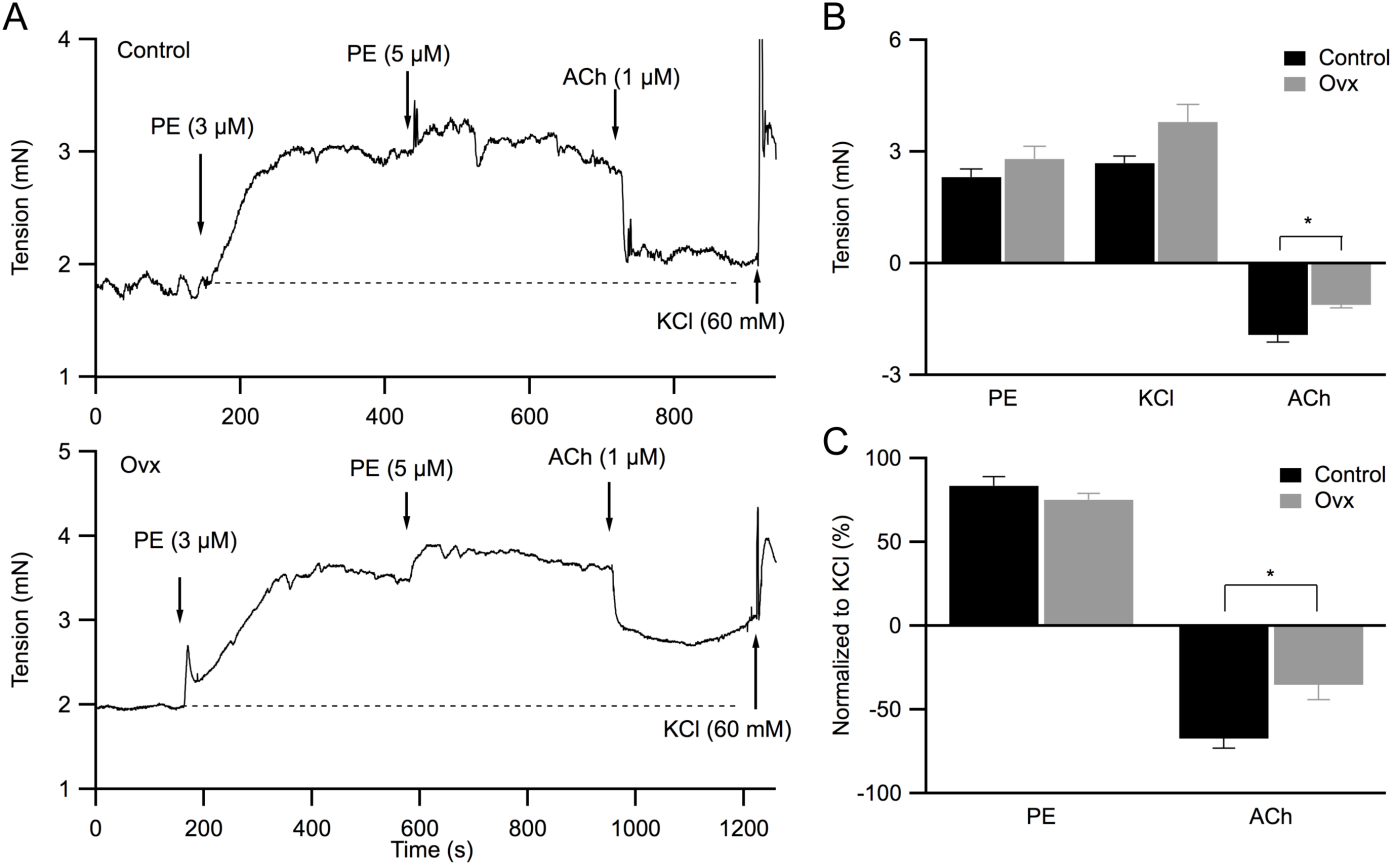
Compromised ACh-induced relaxation in ovx mesenteric vessels. **A**: Representative force myograph traces showing isometric tension (mN) plotted against the time of recording (s) using control (top panel) and ovx (bottom panel) mesenteric vessels. Bath application of PE (3 and 5 µM) induced vasoconstriction, ACh (1 µM) induced vasorelaxation, was followed by bath solution replacement with physiological salt solution containing 60 mM KCl that induced vasoconstriction. **B**: Summary of isometric tension changes induced by PE (3 uM), KCl, and ACh for both control (black) and ovx (grey) vessels. Negative values show vasorelaxation. **C**: Tension changes normalized to the KCl-induced contraction for both PE and ACh. Ovx vessels show reduced ACh response. Asterisk (*) denotes statistical significance (P<0.05, *t*-test).

To further characterize the contractility difference in control and ovx vessels, in a different set of experiments we quantified the effect of both PE- and ACh-induced changes in vascular tension and normalized them to the maximal tension induced with high [K^+^]_o_. Bath applied PE increased the isometric tensions in both control and ovx mesenteric arteries to a similar extent (3 µM PE: control: 2.3±0.2 mN, n = 9; ovx: 2.8±0.3 mN; n = 8; P>0.05; Fig. 2A and B). Subsequent bath applied 1 µM ACh reduced vascular tension was more pronounced in control vessels (1.9±0.2 mN, n = 9) than in ovx vessels (1.1±0.1 mN, n = 8; P<0.05), consistent with a reduced maximal ACh-induced vasorelaxation in ovx vessels (Fig. 1D). Next, bath solution was then replaced with a PSS containing high KCl (increased by 60 mM with 1∶1 KCl:NaCl replacement), which depolarizes smooth muscle cell membrane potential and activates voltage-dependent L-type Ca^2+^ channels, resulting in maximally increased vascular tension in both groups of vessels (control: 2.7±0.2 mN, n = 9; ovx: 3.8±0.5 mN, n = 8; P>0.05; Fig. 2A and B). KCl-induced contraction was comparable to the maximal tension induced with high [PE] and was used to normalize the vasoactive effects of PE and ACh. Figure 2C shows the changes in tension, normalized to KCl-induced contraction, for both PE-induced contraction and ACh-induced relaxation. PE-induced vasoconstriction was similar in mesenteric arteries isolated from both control and ovx groups; however, ACh-induced vasorelaxation was reduced in ovx arteries (control: 67±6%, n = 9; ovx: 35±9%, n = 8; P<0.05), indicating differential ACh-induced endothelium-dependent vasorelaxation between these two animal groups.

### Differential NO, PGI_2_ and EDH contributions to vasorelaxation in control and ovx mesenteric arteries

Acetylcholine induces endothelium-dependent vasorelaxation via three major pathways: eNOS dependent production of nitric oxide (NO), cyclooxygenase dependent production of prostacyclin (PGI_2_), and SK3/IK1 dependent endothelium-derived hyperpolarization (EDH). To further study the difference in ACh-induced vasorelaxation between control and ovx mesenteric arteries, we used selective inhibitors to block each of the three pathways and quantified both their preexisting activity and contribution to ACh-induced relaxation.

**Figure 3:**
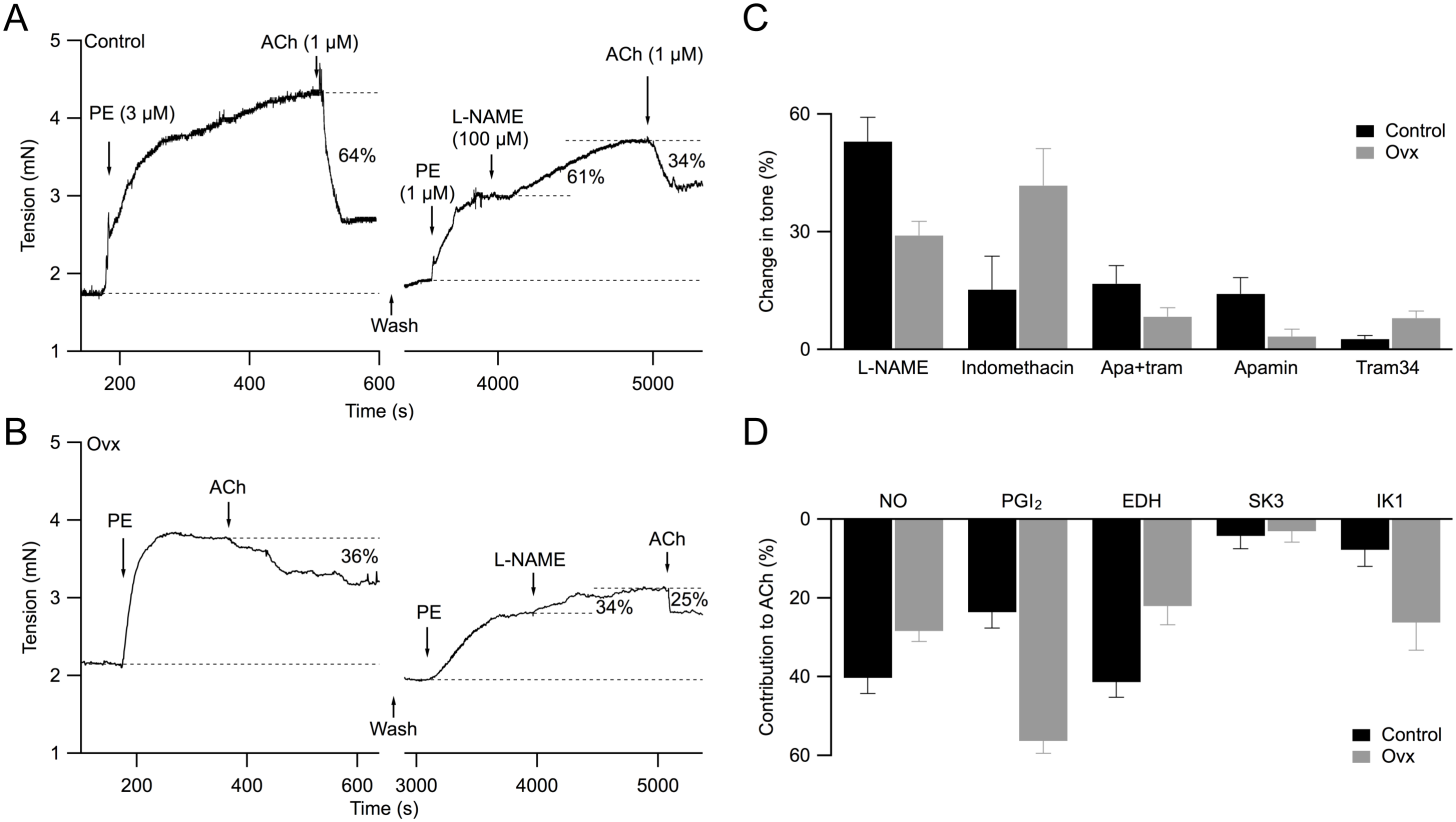
Reduced ACh-induced vasorelaxation due to decreased SK3 channel contribution in ovx vessels. **A**: (left panel) Representative force myograph recording showing tension (mN) plotted against time (s) using a mesenteric vessel obtained from control mouse. Addition of 3 µM PE increased tension and 1 µM ACh caused 64% vasorelaxation, normalized to the PE-induced tension. (right panel) Following bath washout, PE was added to pre-contract the vessel ∼50%, followed by the addition of 100 µM L-NAME and 1 µM ACh. L-NAME-induced 61% increase in PE-induced contraction and ACh reduced tension by 34%. **B**: Representative force myograph trace obtained from an ovx artery. **C and D**: Summarized results for (A and B) and for other selective inhibitors to block different vasorelaxation pathways to study their (**C**) change in tone and (**D**) contribution to ACh-induced relaxation for both control (black bars) and ovx (grey bars) vessels. **C**: Change in tone was obtained from tension increase in the presence of inhibitors normalized to the baseline tension (eg. 61% and 34% increase in the presence of L-NAME for control and ovx vessels, respectively, as shown in A and B). **D**: Contribution to ACh-induced relaxation was calculated from the difference in ACh relaxation before and after inhibitor treatment, normalized to the control (before) ACh relaxation. L-NAME blocks nitric oxide (NO) pathway; indomethacin blocks prostacyclin (PGI_2_) pathway; apamin (apa) and tram34 (tram) together block the EDH pathway.

For these studies, both control and ovx arteries were pre-contracted with PE (3 µM; EC_80_) and subsequently exposed to 1 µM ACh. In control arteries, ACh induced 64% relaxation (Fig. 3A left). Following bath washout, ACh responses were assessed again in the presence of inhibitor. Notably, we reduced the second PE-induced precontraction (1 µM; EC_50_) in order to allow for any additional contractile effects of inhibitors (Fig. 3A right). The representative effect of L-NAME (NG-nitro-L-arginine methyl ester, an inhibitor for NO production) on tone and ACh-induced relaxation in vessels obtained from control mice is shown in Figure 3A (right). Addition of 100 µM L-NAME caused a 61% increase in force (normalized to the force difference between middle and bottom dashed lines as 100%; Fig. 3A right). This increase in force occurred over ∼15–20 min, revealing the presence of NO-dependent activity. Time controls for PE contractions showed less than 14% change in tone over the full course of the functional experiments (∼20 min). This experimental approach revealed the preexistence of NO activity in PE-contracted vessels. Contraction in response to L-NAME suggests regulation of mesenteric arterial tone by nitric oxide even in the absence of direct endothelial stimulation; thus, we attributed this increase to a preexisting activity of NO on vascular tone (summarized in Fig. 3C). In the presence of L-NAME, application of 1 µM ACh induced a 34% reduction in force (of top-to-bottom dashed lines; Fig. 3A right). Thus, ACh-induced vasorelaxation, in the presence of L-NAME, was reduced to 53% (34/64%), indicating that NO contributed 47% to ACh-induced vasorelaxation (summarized in Fig. 3D; see Methods).

Results were also obtained from ovx mouse mesenteric vessels as shown in Figure 3B. In ovx vessels, ACh induced 36% relaxation. Application of 100 µM L-NAME increased force by 34%, and in the presence of L-NAME ACh caused 25% vasorelaxation. Thus in ovx vessels NO contributed 34% to PE-precontracted vascular tone, and 30% (11/36%) to ACh-induced relaxation. Figures 3C and D summarize NO’s preexisting activity (control: 53±6%; ovx: 29±4%; n = 10; P<0.05) and its contribution to ACh-induced relaxation (control: 40±4%; ovx: 28±3%), and indicate diminished NO activity in ovx vessels.

Using the same approach, we further studied the activity of PGI_2_ and EDH to PE-preconstricted tone and their contribution to ACh-induced vasorelaxation (Fig. 3C and D). In the presence of 10 µM indomethacin to block PGI_2_ production, ovx vessels showed an increased PGI_2_ activity (control: 15±8, n = 9; ovx: 42±9; n = 6; P<0.05), and ACh-induced vasorelaxation (control: 24±4; ovx: 56±3). EDH activity and its contribution to ACh-induced vasorelaxation were assessed using apamin and tram34, selective antagonists for SK3 and IK1 channels, respectively. Both SK3 and IK1 channel activity contributes to EDH-mediated vasorelaxation, and blocking these channels together abolishes the EDH pathway [27]. Simultaneous bath application of apamin (300 nM) and tram34 (1 µM) increased the basal force by 17±5% and 8.4±2.3% in control and ovx mice, respectively (n = 7 for both; P>0.05; Fig. 3C), indicating the preexisting EDH activity on vascular tone was only slightly greater in control vessels. Interestingly, the contribution of EDH to ACh-induced vasorelaxation was significantly reduced in ovx vessels compared to controls (control: 41±4; ovx: 22±5; Fig. 3D). Together, our results suggest ovariectomy 1) reduces net ACh-induced vasorelaxation and 2) shifts the contribution of endothelium-dependent vasorelaxation from NO and EDH pathways to PGI_2_ pathway.

### Reduced SK3 channel contribution to EDH-mediated vasorelaxation in ovx vessels

We pharmacologically isolated the individual contributions of SK3 and IK1 channels to EDH-mediated vasorelaxation. Figure 3C summarized the basal channel activity and its influence to vascular tone–blocking SK3 channels alone with 300 nM apamin increased arterial force by 14±4% (n = 6; P<0.05) in control but had little if any effect on ovx vessels (3.3±1.9%; n = 7; P>0.05). Further, blocking IK1 channels alone with 1 µM tram34 had little effect on control and ovx arteries (control: 2.7±0.9; ovx: 8.0±1.8; n = 8; P>0.05; Fig. 3C). The contribution of SK3 and IK1 channels to ACh-induced vasorelaxation is summarized in Figure 3D. Apamin by itself did not significantly affect ACh-induced vasorelaxation in control or ovx arteries (control: 4.2±3.3; ovx: 3.0±2.8; Fig. 3D). Interestingly, tram34 alone had little effect on ACh-induced relaxation in control vessels (8±4%), but its effect was significantly greater in ovx vessels (26±7%; P<0.05). In fact, IK1 inhibition completely abolished EDH-mediated vasorelaxation in ovx vessels. Together, the results suggest 1) both SK3 and IK1 channel activity contributes to EDH-mediated relaxation, 2) blocking only SK3 or IK1 in control vessels has minimal influence on EDH-mediated relaxation, and 3) ovariectomy essentially abolishes SK3 channel but not IK1 channel contribution to EDH-mediated vasorelaxation.

### IK1 channels mediate TRPV4-dependent vasorelaxation in ovx vessels

**Figure 4:**
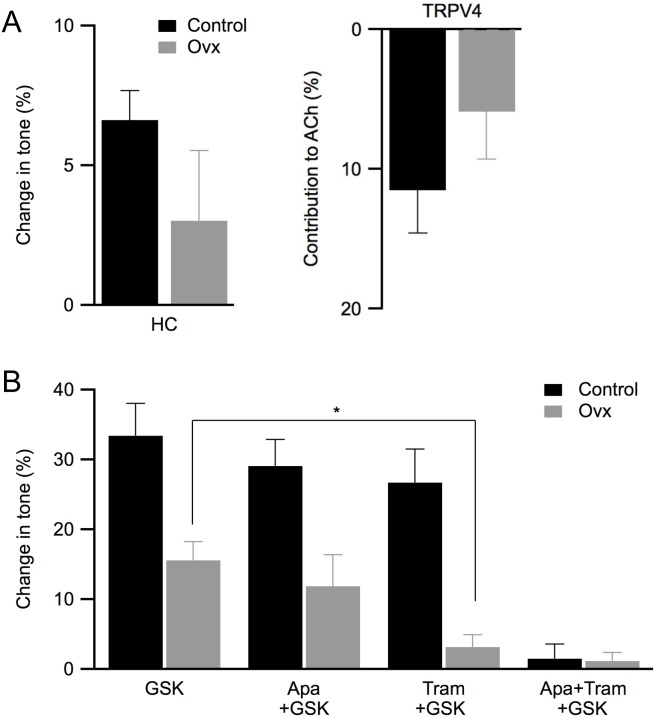
IK1 channel activity mediates TRPV4-induced vasorelaxation in ovx vessels. **A**: Summarized results from studies using 500 nM HC067047 (HC), a TRPV4 channel antagonist, on change in tone (left panel) and contribution to ACh-induced relaxation (right panel) using vessels obtained from both control (black) and ovx (grey) mice. **B**: Summarized results from studies using 300 nM GSK1016790, a TRPV4 channel agonist, on changes to vascular tension in the absence and presence of apamin (apa) and/or tram34 (tram). These studies were performed in the presence of L-NAME and indomethacin. Asterisk (*) denotes statistical significance (P<0.05, *t*-test).

Previous studies have shown that endothelial TRPV4 channels provide Ca^2+^ activation of SK3 and IK1 channels [10], [28]. Thus, we examined whether the ovx-induced shift in IK1/SK3 channel contribution to EDH-mediated vasorelaxation would be reflected by changes in TRPV4 channel activity. We performed the same experiments but in the presence of both 100 µM L-NAME and 10 µM indomethacin to block NO and PGI_2_ pathways, respectively (Fig. 4). In this condition, EDH is the major contributor to vascular tone. Application of HC067047 (HC, 500 nM) to block TRPV4 channels modestly increased basal force (control: 6.6±1.1; ovx: 3.0±2.5; n = 8; P>0.05; Fig. 4A left) and reduced ACh-induced vasorelaxation (control: 11.5±3.1; ovx: 5.9±3.4; P>0.05; Fig. 4A right) in both groups, consistent with contribution of TRPV4 channel activity to vasorelaxation.

We then tested the TRPV4 channel agonist GSK1016790 (GSK) in the presence or absence of SK3 and/or IK1 blockers to determine whether vasorelaxation induced by TRPV4 channel activation: 1) requires SK3 and/or IK1 channel activity, and 2) reflects increased dependence on IK1 channel activity in ovx vessels. In the presence of L-NAME and indomethacin to isolate EDH-mediated vasorelaxation, 300 nM GSK-induced vasorelaxation was more prominent in control than ovx vessels (control: 33±5% vs. ovx: 16±3%; n = 8; P<0.05; Fig. 4B). In a separate control study GSK induced vasorelaxation was abolished in the presence of HC, consistent with the involvement of TRPV4 channels (data not shown). While GSK induced vasorelaxation in control vessels was unaffected by apamin or tram34 alone, it was completely abolished by the combination of apamin and tram34 (apa+tram; Fig. 4B), indicating the TRPV4-induced vasorelaxation was dependent upon the activity of both SK3 and IK1 channels (Fig. 4B). Notably, GSK induced vasorelaxation was reduced in ovx vessels compared to controls. This remaining vasorelaxation in ovx was entirely dependent upon IK1 channel activity as it was blocked by tram34 or tram34+apamin but was unaffected by apamin alone (for tram+gsk: control: 27±5%; ovx: 3.2±2%; n = 6; P<0.05; Fig. 4B). These results are consistent with functionally coupled TRPV4, IK1, and SK3 channels whereby activating TRPV4 channels results in both IK1 and SK3 channel activation, leading to EDH-mediated vasorelaxation. In control vessels IK1 and SK3 channel activity compensates for one another; however, in ovx vessels TRPV4 channel activation-induced vasorelaxation is diminished possibly due to reduced SK3 channel activity, rendering it more dependent on IK1 channel activity. Taken together, results from these functional studies suggest that ovariectomy reduces functional coupling between TRPV4 and SK3 channels, leading to a reduction in the contribution of SK3 channels to vasorelaxation. These results are consistent with a model in which ovariectomy reduces endothelial SK3 channel activity, leading to reduced contribution of SK3 channel activity to ACh-induced vasorelaxation.

### Whole cell recording of SK3 and IK1 current density from mesenteric artery endothelial cells

**Figure 5:**
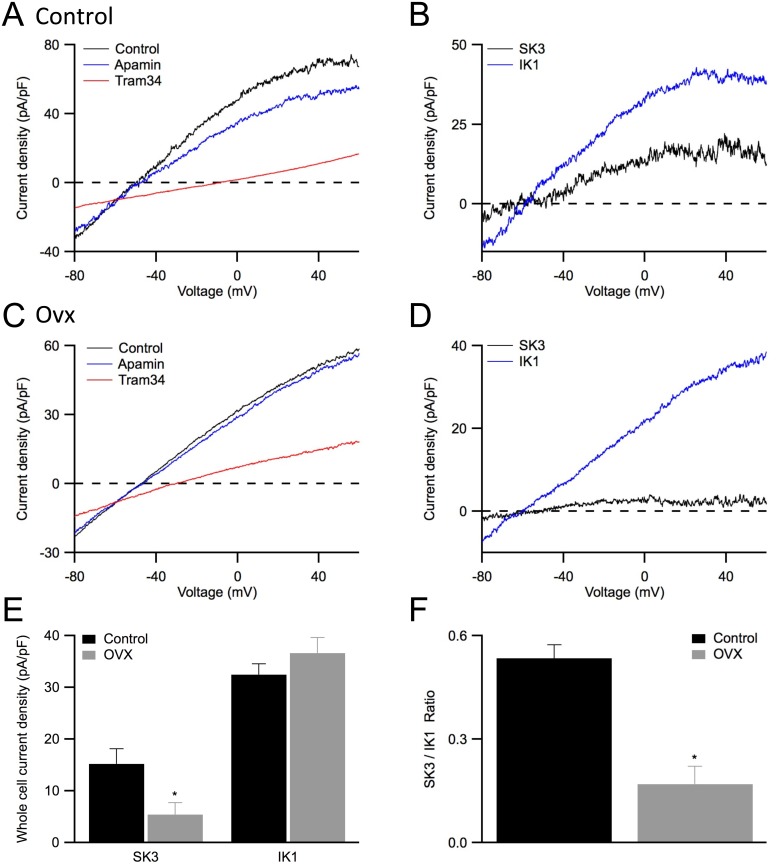
Ovariectomy reduces SK3 channel current density in endothelial cells. **A**: Representative traces recorded using conventional whole-cell recording on endothelial cells isolated from mesenteric arteries obtained from control mouse. Cells were voltage clamped at their resting membrane potential and a 200 ms voltage ramp from of −80 to +60 mV was delivered to elicit whole cell currents before (control) and after subsequent bath application of apamin (apamin) and apamin+tram34 (tram34). **B**: SK3 and IK1 current densities isolated from digital subtraction of the traces shown in (A) for control endothelial cells. **C**: Representative whole-cell current density obtained from ovx endothelial cells. **D**: SK3 and IK1 current densities isolated from digital subtraction of the traces in (C) for ovx endothelial cells. **E**: Summarized whole-cell SK3 and IK1 current densities from control (black) and ovx (grey) endothelial cells measured at +30 mV. **F**: Normalized SK3/IK1 ratios for control (black) and ovx (grey) recordings showing reduced SK3 channel activity in ovx endothelial cells. Asterisk (*) indicates statistical significance from control (P<0.05, *t*-test).

To directly test the hypothesis that ovariectomy reduces endothelial SK3 channel activity, we performed whole-cell patch clamp recordings. Vascular endothelial cells (ECs) were acutely dispersed from first- and second-order mouse mesenteric arteries. ECs were visually identified by their characteristic phase contrast under light microscopy as previously reported [7]. Whole-cell voltage clamp protocols were performed in the presence of 3 µM free internal Ca^2+^ to activate SK3 and IK1 channels and currents were elicited with a 200 ms voltage ramp from −80 to +60 mV, delivered every 30 seconds. We obtained current densities by normalizing recorded whole-cell currents to the membrane capacitance, calculated from a hyperpolarizing step (Fig. 5A and C). Whole cell current density averages measured at +30 mV for control and ovx ECs were 68±11 pA/pF (n = 10) and 55±13 pA/pF (n = 7; P>0.05), respectively. Bath applied apamin (300 nM) reduced current density and the SK3 current density was isolated by digital subtraction for both control and ovx ECs (Fig. 5B and D, respectively). Ovx ECs showed reduced SK3 current density (control: 15±2.9 pA/pF; ovx: 5.5±2.3 pA/pF; n = 6; P<0.05; Fig. 5E). Subsequent addition of tram34 (1 µM) further reduced whole-cell current density (Fig. 5A and C); however, digitally isolated IK1 current density was not different between control and ovx ECs (control: 32±2.1 pA/pF; ovx: 37±3.0 pA/pF; n = 6; P>0.05; Fig. 5B, D, and E). We further calculated the normalized contribution of SK3 and IK1 channels to EC current density and plotted the ratios of SK3/IK1 for both control and ovx ECs. Consistent with our functional studies, the significantly higher SK3/IK1 ratio in control ECs showed a 3-fold increase in SK3 channel activity as compared to that of ovx ECs (control: 0.54±0.04; oxv 0.17±0.05; P<0.05; Fig. 5F).

### Reduction in GSK-sensitive TRPV4 channel current density in ovariectomized mice

**Figure 6:**
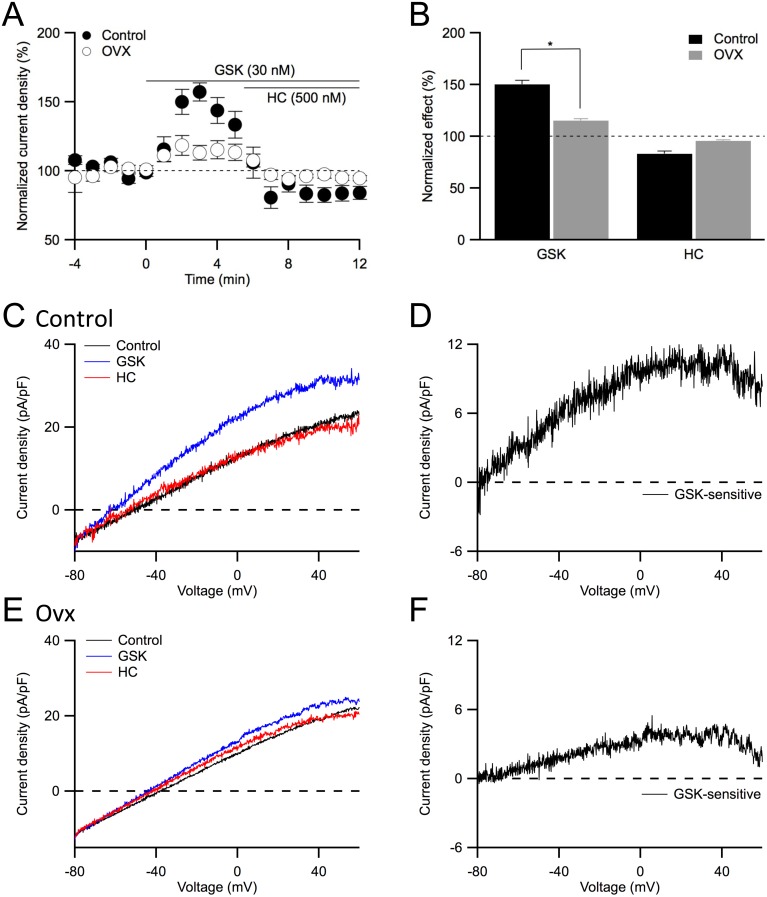
TRPV4 channel activation evokes a smaller response in ovx endothelial cells. **A**: Time course of the whole-cell current density evoked at +30 mV from control (solid) and ovx (open) endothelial cells using perforated patch clamp technique. Following a stable baseline, 30 nM GSK1016790 (GSK, 0 min) was added to the bath to activated TRPV4 channels, followed by bath application of 500 nM HC067047 (HC, 6 min) to block them. Time course was normalized to baseline. **B**: Averaged current density in the presence of GSK (3–5 min as shown in A) and GSK+HC (9–11 min), normalized to the control current density (−3 to −1 min). Asterisk (*) indicates statistical significance (P<0.05, *t*-test). **C**: Representative traces recorded from an endothelial cell obtained from control mesenteric artery at different conditions as shown in (A), before (control) and after subsequent bath addition of GSK (GSK) and GSK+HC (HC). Whole cell current density was elicited with −80 to +60 mV voltage-ramps. **D**: GSK-sensitive whole-cell current density isolated from digital subtraction of the traces (C) for control cells. **E**: Representative perforated whole-cell current density obtained from ovx endothelial cells. **F**: GSK-sensitive current density isolated from digital subtraction of the traces (E) for ovx cells.

Results thus far suggest that the reduced SK3 channel activity underlies reduced EDH-mediated vasorelaxation in ovx vessels. This model predicts that activation of TRPV4 channels with GSK should induce a smaller SK3 channel-mediated current in ovx ECs. We tested this hypothesis using perforated whole-cell recordings, in which the intracellular [Ca^2+^] remained intact and the calcium activation of endothelial Ca^2+^-activation potassium (K_Ca_) channels was examined. Perforated whole-cell current density was elicited using the same whole-cell voltage ramp protocol as described in Figure 5, and TRPV4 channels were activated with bath application of GSK. Changes in current density, due to TRPV4 channel activation, were quantified using the steady-state current density elicited at +30 mV and normalized to the baseline before GSK (Fig. 6A). Following a stable baseline recording, bath applied GSK (30 nM) increased the whole-cell current density of ECs isolated from control mice to 150±4% (n = 6; Fig. 6B). In contrast, the effect of GSK on whole-cell current density was reduced in ovx ECs (ovx: 116±2%; n = 6; P<0.05; Fig. 6A and B). Subsequently bath applied HC (500 nM) reduced the current density to 84±5% (control) and 96±3% (ovx) of baseline (n = 6; P>0.05). The whole-cell current density, induced by activation of TRPV4 channels and digitally isolated for control (Fig. 6C and D) and ovx ECs (Fig. 6E and F), reversing at very negative membrane potentials resembling K^+^ currents, suggested TRPV4-dependent activation of K_Ca_ channels. Notably, activation of TRPV4 channels caused a smaller increase in K_Ca_ current density in ovx ECs (Fig. 6B). Taken together, these results suggest TRPV4-induced SK3 channel activation is significantly reduced in ovx ECs due to reduced SK3 current density, resulting in a reduced EDH-mediated vasorelaxation in mesenteric vessels obtained from ovx mice.

## Discussion

Female hormones naturally produced by the ovaries have been shown to be beneficiary to the wellbeing of women. Nonetheless, following either natural menopause or prophylactic surgical removal of ovaries against ovarian cancer, women not only lose the protective benefits of circulating ovarian hormones, but may also develop higher risk factors for diseases compared to age-matched women with intact ovaries. One such example is the loss of cardiovascular protection following natural menopause or ovariectomy (ovx), increasing risk factors for cardiovascular diseases such as coronary and atherosclerotic disease [29], [30]. Consistent with previous findings, we show reduced endothelium dependent vasodilation capacity in mesentery arteries of ovariectomized mice [15]. We further show that the normally high dependence on endothelium-derived NO and EDH relaxation pathways shifts to a predominant role for PGI_2_ following ovariectomy. Furthermore, the reduced EDH contribution to vasorelaxation in ovx vessels is due to a reduction in SK3 channel activity and a corresponding decreased response to TRPV4 channel activation.

Vascular endothelial IK1 and SK3 channels have been shown to modulate vascular tone via a NO- and PGI_2_-independent pathway [31]. Activation of these endothelial K_Ca_ channels results in smooth muscle cell hyperpolarization, and is hence referred to as endothelium-dependent hyperpolarization (EDH) of smooth muscle cells. EDH, an important modulator of vascular tone, is dependent upon the vessel size [32], aging [33], diabetes [34], and circulating hormones [35]. The exact mechanism underlying EDH is still not fully understood because many factors converge and contribute to the membrane hyperpolarization phenomenon. Studies have shown that EDH could be achieved through direct electrical coupling between endothelial and smooth muscle cells, accumulation of K^+^ ions around smooth myocytes, or release of other vasorelaxation factors other than NO or PGI_2_ that causes membrane hyperpolarization (see review [36]). Importantly, these mechanisms converge and the activation of endothelial K_Ca_ channels leads to smooth muscle hyperpolarization and vessel relaxation [37]. We and others have previously reported that the K_Ca_ channels in systemic endothelial cells consist of IK1 and SK3 channels [5], [7]. Recent studies have unraveled the interplay between endothelial K_Ca_ channels with TRPV4 channels; activation of TRPV4 channels allows Ca^2+^ influx, which either directly or through enhanced Ca^2+^ release from intracellular stores, activates SK3 and IK1 channels, leading to membrane hyperpolarization [10], [28].

Previous studies have shown that both SK3 and TRPV4 channels colocalize in endothelial caveolae via their interaction with the structural caveolar proteins, caveolin-1 [8], [38]. Caveolae are specialized mobile vesicles that serve as hubs for signaling complexes. They have been shown to modulate trafficking, surface expression, and activity of both SK3 and TRPV4 channels. For example, we have shown that aortic endothelial SK3 channel trafficking is activated by an increased [Ca^2+^]_i_, which can be induced with bath applied ACh [7]. Furthermore, TRPV4 channel trafficking and surface expression are modulated both by myosin light chain kinase activity, and by forming heteromeric channels with TRPC1 isoform that directly interacts with caveolin-1 [39]–[41]. In contrast, ubiquitination modulates IK1 channel trafficking via the lysosomal pathway, which does not utilize caveolar trafficking mechanisms [42], [43]. Despite the difference in subcellular localization and trafficking of SK3 and TRPV4 channels from IK1 channels, our functional studies show that both SK3 and IK1 channels contribute directly to ACh-induced vasorelaxation (Fig. 3). Similarly, TRPV4 channel activation signals through both SK3 and IK1 channels because inhibiting either SK3 or IK1 alone does not reduce GSK-induced EDH vasorelaxation whereas their co-inhibition abolishes it (Fig. 4). SK3 and IK1 channels function in a compensatory fashion to contribute to ACh-induced relaxation, TRPV4-dependent changes in K_Ca_ currents, and EDH-mediated vasorelaxation, consistent with functional coupling of endothelial TRPV4, SK3 and IK1 channels. [10].

In addition to SK3 and TRPV4, eNOS (endothelial nitric oxide synthase) also binds to caveolin-1, which inhibits its activity to produce NO [44]. Our results indicate that ovariectomy reduces the basal activity of NO and EDH, as well as their contribution to vasodilation. Although the mechanism underlying ovariectomy-dependent reduction in NO was not the focus of this study, we showed that the contributions of NO to PE-induced tone and to ACh-dependent vasorelaxation were both reduced in ovx vessels. The effects of estrogen on the endothelial NO pathway seem to vary among animal species, vascular bed, and vessel size [2], [15], [22]. Specifically, estrogen has been shown to both up- and down-regulate eNOS activity via numerous mechanisms, including changes in caveolin expression, gap junction expression, intracellular Ca^2+^ handling, and even SK3/IK1 channel activity [16], [45]–[48]. Moreover, ovx reduces endothelial SK3 current density, which consequently reduces the effect of TRPV4 channel activation-induced basal relaxation, and shifts EDH-mediated relaxation to be more dependent upon IK1 channel activity. While it is possible that reduced gap junctions may explain the decreased EDH vasorelaxation [16], our electrophysiological recordings from isolated cells further show reduced SK3 current density. Because caveolin-1 proteins are crucial for the formation of caveolae and their scaffolding domain also form signaling microdomains, a possible explanation is that ovx enhances the expression of caveolin-1, and consequently downregulates eNOS and TRPV4/SK3 channel activity [7], [44], [49]. This could lead to reduced NO and SK3 dependent vasorelaxation while preserving IK1 contribution.

It is also possible that ovx reduces endothelial SK3 channel expression. Ovariectomy-induced downregulation of SK3 channel mRNA expression has been reported in different regions of guinea pigs’ brains, and the effect is attributable to the levels of circulating estrogen [50]. Upon further dissection and separation of different neurons, the same group recently showed that loss of estrogen may upregulate SK3 channel mRNA expression in a subpopulation of GnRH neurons of the hypothalamus [21]. Thus, estrogen regulation of SK3 channel expression could depend upon the specific cell types and transcription factors associated with the estrogen receptor [20]. Our results that show whole-cell SK3 channel current density is reduced following ovx could also be a consequence of estrogen-dependent loss of SK3 channel expression, and future studies with estrogen replacement will be required to address this. In contrast, IK1 channel current density was unchanged between control and ovx ECs. Indeed a downregulation of SK3 channel expression in ovx vessels would be consistent with the findings of our functional studies. That is to say, EDH vasorelaxation requires both SK3 and IK1 channels in control vessels but is mediated only by IK1 channels in arteries from ovx mice (Fig. 3D). Complete evaluation of endothelial SK3 expression levels and distribution profiles following ovariectomy is warranted. Regardless of mechanism, it appears EDH-mediated relaxation becomes highly dependent upon IK1 channel activity following ovariectomy due to the reduction of endothelial SK3 channel activity.

The TRPV4 results obtained using myography are consistent with that from perforated whole-cell recordings. Our functional studies show that in control vessels GSK-induced vasorelaxation is mediated via either SK3 or IK1 channels, indicating that activating TRPV4 channels would activate both SK3 and IK1 channels. Perforated recordings also show the GSK-induced increase in current density is reversible by HC067047, a selective TRPV4 antagonist, suggesting the changes in current density is induced by changes in TRPV4 channel activity. Indeed the currents isolated using digital subtraction show characteristic outward K^+^ currents reversing at negative potentials. Interestingly, perforated K_Ca_ current density activated by GSK being significantly reduced in ovx ECs was surprising because IK1 channel activity does not fully compensate for the loss of SK3 channel activity. This could simply reflect reduced SK3 current density or it could implicate that TRPV4 channel activation either does not activate all K_Ca_ channels or does not fully activate them in ovx ECs. It is also possible that ovx may cause uncoupling of IK1 channels from TRPV4 channels or reduction of the Ca^2+^ sensitivity of IK1 channels. Additional studies are required to examine possible causes of this disparity, including issues related to [Ca^2+^]_i_ stimulated by GSK, Ca^2+^ sensitivity of IK1 channels, and subcellular expression profiles of IK1 channels in control and ovx endothelial cells.

In conclusion, ovariectomy induces significant changes in endothelium-dependent vasorelaxation in murine mesenteric arteries. The loss of circulating ovarian hormone(s) reduces ACh-induced vascular relaxation by shifting from NO- and EDH-mediated toward PGI_2_-mediated vasorelaxation. The reduced EDH relaxation in vessels obtained from ovx animals is likely due to reduced endothelial SK3 channel activity. Correspondingly, both TRPV4 activation-induced vasorelaxation and activation of whole-cell current density are reduced in ovx as compared to that of control. On the other hand, IK1 channel activity remains similar between the two animal groups, suggesting that while SK3, IK1, and TRPV4 channels are functionally coupled in the mesenteric artery endothelium, ovariectomy disrupts the functional coupling of TRPV4 and IK1 channels. Consequently, the reduced SK3 activity and functional uncoupling between TRPV4 and IK1 channels following ovariectomy result in a reduced EDH-mediated vasorelaxation.
